# Multisystem clinicopathologic and genetic analysis of MELAS

**DOI:** 10.1186/s13023-024-03511-4

**Published:** 2024-12-24

**Authors:** Shuai Xu, Jialiu Jiang, Leilei Chang, Biao Zhang, Xiaolei Zhu, Fengnan Niu

**Affiliations:** 1 Department of Neurology, Nanjing Drum Tower Hospital, Affiliated Hospital of Medical School, Nanjing University, Zhongshan Road 321#, Nanjing, 210008 Jiangsu China; 2 Department of Pathology, Nanjing Drum Tower Hospital, Affiliated Hospital of Medical School, Nanjing University, Zhongshan Road 321#, Nanjing, 210008 Jiangsu China

**Keywords:** MELAS, MRS, Stroke-like episodes, RRFs, COX enzyme defects

## Abstract

**Background and objectives:**

Mitochondrial encephalomyopathy with lactic acidosis and stroke-like episodes (MELAS) syndrome is a maternally inherited mitochondrial disorder that mostly affects the central nervous system and skeletal muscle. This study provides a comprehensive summary of the clinical symptoms, multisystemic pathogenesis, and genetic characteristics of MELAS syndrome. The aim was to improve comprehension of clinical practice and gain a deeper understanding of the latest pathophysiological theories.

**Methods:**

The present investigation involved a cohort of patients diagnosed with MELAS at Nanjing Drum Tower Hospital between January 2014 and December 2022. Multisystem symptoms, magnetic resonance imaging/spectroscopy (MRI/MRS), muscle biopsy, and mitochondrial DNA (mtDNA) data were summarized and subsequently analysed.

**Results:**

This retrospective study included a cohort of 29 MELAS patients who predominantly presented symptoms such as stroke-like episodes, proximal muscle weakness, and exercise intolerance. MRI scans revealed very small infarcts beneath the deep cortex during stroke-like episodes, indicating nonvascular brain damage. Pathology analyses of the brain also showed neuronal degeneration and glial cell proliferation in the cerebral parenchyma. Proton magnetic resonance spectroscopy (^1^H-MRS) analysis revealed an increase in the lactate peak and a reduction in the N-acetylaspartate (NAA) level. Similarly, the phosphorus magnetic resonance spectroscopy (^31^P-MRS) analysis revealed an abnormal ratio of inorganic phosphate (Pi) to phosphocreatine (PCr). Muscle biopsy revealed the presence of ragged red fibres (RRFs) and cytochrome c oxidase (COX) enzyme-defective cells. These abnormalities indicate structural abnormalities in the mitochondria and deficiencies in oxidative phosphorylation, respectively. In addition to the common m.3243A > G variant, other prevalent variants, including m.5628 T > C, m.6352-13952del, and a 9-bp small deletion combined with m.3243A > G, exist.

**Conclusions:**

MELAS is a rare mitochondrial syndrome characterized by clinical heterogeneity and genetic heteroplasmy. Abnormalities in mitochondrial metabolic function and impairments in enzyme activity are the pathogenic processes underlying MELAS. Mitochondrial vasculopathy and mitochondrial neuropathy may provide a partial explanation for the unique aetiology of stroke-like episodes.

**Supplementary Information:**

The online version contains supplementary material available at 10.1186/s13023-024-03511-4.

## Background and objectives

Mitochondrial encephalomyopathy, lactic acidosis, and stroke-like episode (MELAS, OMIM #540,000) syndrome is a common subtype of mitochondrial encephalomyopathy, characterized by maternal inheritance. The pathogenic mechanism of MELAS is the mitochondrial DNA variant (m.3243A > G) in the mitochondrial leucine tRNA-1(*MT-TL1*) gene encoding tRNA^Leu^-(UUR), which accounts for approximately 80% of MELAS patients [1]. The variant at position 3243 of the mtDNA replaces adenine with guanine and causes a decrease in mitochondrial activity and a reduction in adenosine triphosphate (ATP) synthesis, which in turn leads to malfunction in multiple organs. MELAS primarily affects the central nervous system and skeletal muscle, resulting in various clinical symptoms such as stroke-like episodes, seizures, migraine-like headaches, ophthalmoplegia, proximal muscle weakness, hearing impairment, diabetes mellitus, and short stature [2–4].

The diagnosis of MELAS is mostly based on histological and molecular evidence. Muscle biopsy revealed the presence of ragged red fibres (RRFs), which suggests an aberrant increase in mitochondrial production, as well as the presence of cytochrome c oxidase (COX)-positive or -negative fibres, which indicates a malfunction in mitochondrial enzyme activity. In addition, magnetic resonance imaging/spectroscopy (MRI/MRS) also plays a vital role in the diagnosis of MELAS. Proton magnetic resonance spectroscopy (^1^H-MRS) can identify aberrant brain lesions and metabolic abnormalities, such as increased lactate (Lac) levels and decreased N-acetylaspartate (NAA) concentrations [5]. Phosphorus magnetic resonance spectroscopy (^31^P-MRS) can assess abnormalities in intracellular phosphorus metabolism and evaluate energy metabolism at ATP levels, along with the concentrations of phosphocreatine (PCr) and inorganic phosphate (Pi) (often denoted as Pi/PCr) [6].

The clinical manifestations of MELAS may vary during the initial phases of the disease and could be confused with other disorders, such as acute ischaemic stroke, encephalitis, and epilepsy. Hence, a comprehensive evaluation that encompasses clinical symptoms, familial background, laboratory tests, and histological or genetic findings plays a crucial role in achieving an early and accurate diagnosis, thereby preventing any incorrect or overlooked diagnoses. The relationships between clinical symptoms and underlying physiological processes are not completely understood. This study provides a summary of the clinical and pathological features, imaging examinations, and genetic testing results of 29 patients diagnosed with MELAS syndrome. The purpose of this study was to improve the understanding of this rare mitochondrial disease and to assist in the diagnostic process.

## Materials and methods

### Patient selection

This retrospective investigation included 29 individuals who were diagnosed with definite MELAS syndrome and admitted to the Affiliated Drum Tower Hospital of Nanjing University Medical School between January 2014 and December 2022. The diagnosis of MELAS syndrome was based on the diagnostic criteria established by Shuichi Yatsuga et al. [7], which necessitate the fulfilment of a minimum of two A-class criteria (headache with vomiting, epileptic seizures, hemiplegia, cortical blindness, and acute focal lesions on neuroimaging) and two B-class criteria (elevated plasma or cerebrospinal fluid lactate levels, RRFs in muscle biopsy, and m.3243A > G gene variant). This study was approved by the Medical Ethics Committee of the Affiliated Drum Tower Hospital of Nanjing University Medicine School, and all individuals provided written informed consent.

### Clinical data

The clinical data were collected from both outpatient visits and hospital records, including information such as sex, age, clinical symptoms, and illness progression. To guarantee a precise evaluation of the patients' medical background, comprehensive information was documented regarding the period of symptom onset, early symptoms, primary clinical manifestations, family history, and previous medical history. A scatter plot was generated using R software (version 4.3.1) to display the relationship between survival and mortality. To protect patient privacy and ensure data accuracy, we strictly followed medical ethical standards and obtained informed consent from patients during the data collection process. All the data were anonymized to ensure personal identity confidentiality and data security.

### Imaging (MRI and MRS) examinations

MRI and MRS were conducted to evaluate brain lesions, cerebral abnormalities, and metabolic irregularities. MRI scans were conducted with a 3.0 T magnetic field strength. The scanning sequences included T1-weighted imaging (T1WI), T2-weighted imaging (T2WI), fluid-attenuated inversion recovery (FLAIR), and diffusion-weighted imaging (DWI) sequences. The study adopted a 3.0 T scanner (Achieva 3.0 T TX; Philips Medical Systems, Eindhoven, Netherlands) for ^1^H-MRS. The MRS examination process consisted of the following steps: (1) Placing spectral grids via specific parameter settings in T2-weighted sequences; (2) performing MRS examination via a two-dimensional point source deconvolution spectrum (PRESS) pulse sequence; (3) localizing the field of view (FOV) on a transverse T2-weighted image and selecting voxels from relative regions of the lesion and brain for further analysis; (4) optimizing local magnetic field uniformity via the pen-shaped beam smoothing algorithm (PB-1000 automatic); (5) optimizing local magnetic field uniformity via the pencil beam shimming algorithm (PB-auto); and (6) applying water suppression in excitation mode. Spectral measurements of lactate (Lac), N-acetylaspartate (NAA), total choline (tCho), myo-inositol (mI), and creatine (Cr) were performed via the extended workspace method (EWS; Philips Medical Systems, Eindhoven, Netherlands). Metabolite intensity ratios were calculated using Cr as an internal reference metabolite. The ^31^P-MRS technique utilized a 3.0 T MRI scanner (Achieva 3.0 T TX; Philips Medical Systems, Eindhoven, Netherlands). The subject was evaluated through periods of rest, exercise, and recovery. During the exercise phase, a load equal to 25% of the maximum voluntary contraction was applied to the right ankle. The patient was then directed to kick the ankle to contract the quadriceps. Spectral measurements of inorganic phosphate (Pi), phosphocreatine (PCr), α-ATP, β-ATP, and γ-ATP were obtained via a 50-mm-diameter surface coil positioned on the quadriceps femoris. Each spectrum was scanned for 50 s and analysed using a post processing workstation (EWS, Philips Medical Systems). The ^31^P-MRS investigation consisted of three phases: resting/baseline measurements, dynamic/active loading, and recovery measurements. The ratios of Pi/PCr and ATP/PCr were calculated for each phase.

### Genetic analysis

Genomic analysis was conducted on a cohort of 29 individuals. Peripheral blood or urine samples were collected for DNA extraction and purification. Total genomic DNA was extracted a TIANamp Genomic DNA Kit (DP304; TIANGEN; Beijing; China) to detect the patient's genotype via polymerase chain reaction (PCR) amplification and sequencing. To identify MELAS syndrome, variant sites on mitochondrial DNA were identified by comparing and evaluating the acquired sequences with standard sequences. All the subjects in our study underwent mtDNA sequencing. Twenty-three patients underwent Sanger sequencing for point variant detection at positions 3243 and 8344 (the most common variant positions of mitochondrial encephalomyopathy). Six patients underwent next-generation sequencing, including full-length mitochondrial DNA (mtDNA) sequencing and nucleic DNA(nDNA) sequencing. The molecular investigations of next-generation sequencing included point variants (both mtDNA and nDNA), large-scale deletions and repetitive variants (mtDNAs).

### Histological examinations

A total of 16 patients underwent muscle biopsy, in which their biceps brachii muscle or lateral thigh muscle biopsied while under local anaesthesia. The biopsy samples were frozen in liquid nitrogen isopentane and sectioned at a thickness of 8–10 μm for histological staining, including hematoxylin and eosin (H&E), nicotinamide adenine dinucleotide hydrogen (NADH), cytochrome C oxidase (COX), succinate dehydrogenase (SDH), modified Gomori trichrome (MGT), periodic acid-Schiff (PAS), and Oil Red O staining. The frozen sections were rapidly fixed in a 4% formaldehyde solution for three minutes and then subjected to immunohistochemical/immunofluorescence staining using a Ventana Benchmark XT instrument (Ventana Medical Systems Inc., Tucson, AZ, USA) following established protocols. The anti-mitochondrion antibody (AMA) (clone MTC02, Abcam ab3298) was utilized at a dilution of 1:200 to identify the aberrantly proliferating mitochondrial RRFs. This method served as a complementary approach to the H&E and MGT staining techniques. Alexa Fluor Plus 594, a common red fluorescent dye, was used as a conjugated secondary antibody, whereas 4',6-diamidino-2-phenylindole (DAPI) was utilized to label the cell nuclei. In addition, electron microscopy analysis was performed on 10 samples. The muscle tissues were fixed with a 2.5% glutaraldehyde solution, with a volume of approximately 0.3*0.2*0.1 cm^3^. A suspected patient with a left frontotemporal lobe lesion underwent a surgical procedure to excise a portion of the brain tissue due to an initial misdiagnosis of the neoplasm. This brain tissue was examined via paraffin-embedded sections. Another suspected patient with impaired kidney function characterized by the presence of proteinuria underwent renal biopsy as a result of an initial incorrect diagnosis of nephropathy. The kidney samples were processed into frozen sections and examined via COX, SDH and NADH enzyme activity staining.

## Results

### Clinical characteristics

**Fig. 1:**
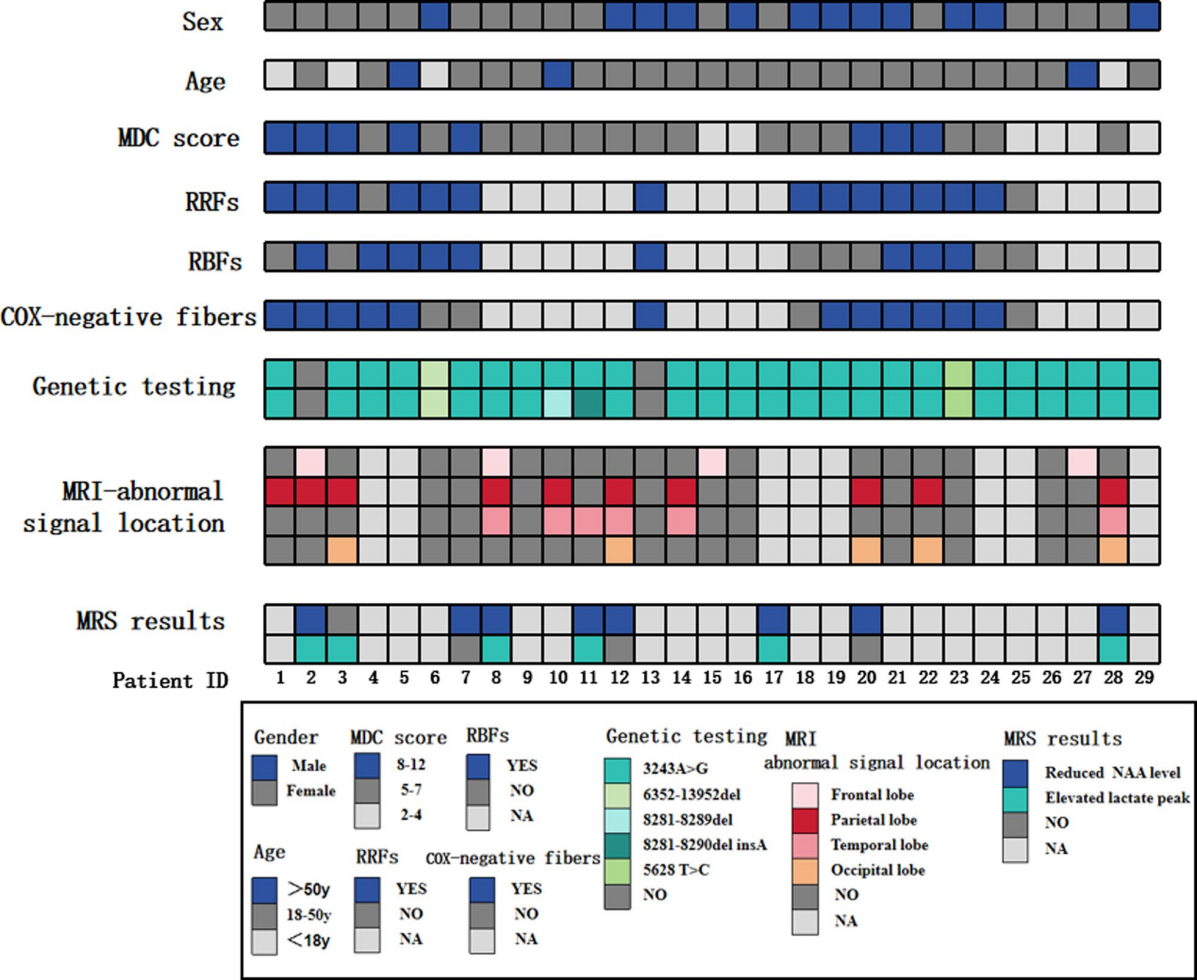
Statistical features and diagnostic examination results of 29 patients with MELAS syndrome.The grid displays the details of 29 MELAS patients, including sex, age, MDC score, presence of RRFs, RBFs, COX-negative fibres, genomic analysis results, and different sites of lesions observed on MRI (frontal lobe, parietal lobe, temporal lobe, and occipital lobe). Each piece of information is represented by a different colour, as shown in the figure

**Fig. 2:**
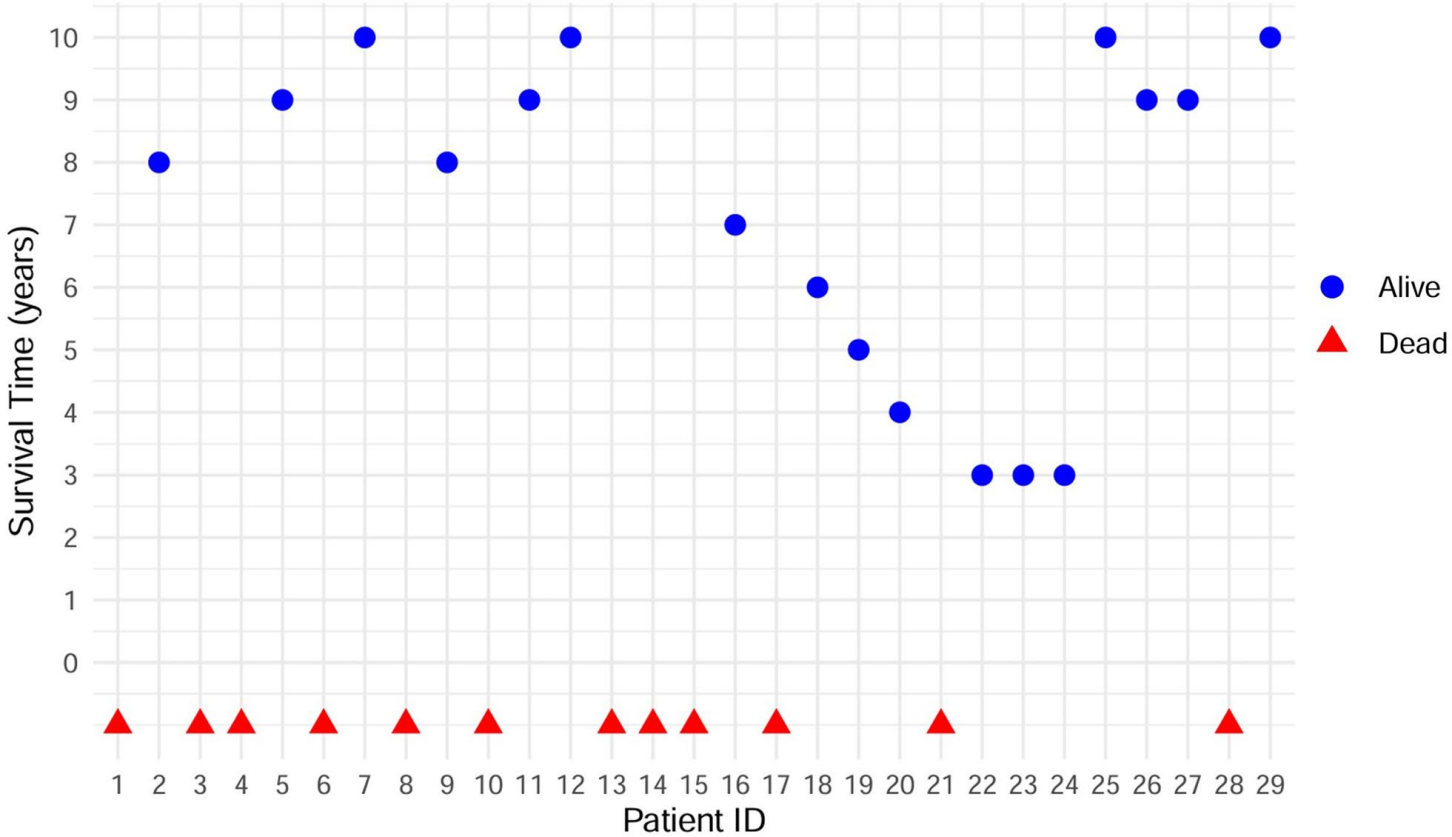
Scatter plots depicting the survival and mortality outcomes of 29 patients diagnosed with MELAS syndrome. The x-axis (horizontal) depicts the case number, whereas the y-axis (vertical, above zero) denotes the duration of survival from the diagnosis of MELAS syndrome to the current year. The red triangles indicate patients who have passed away, whereas the blue dots reflect those who are still alive

In our group of 29 MELAS patients, the male-to-female ratio was 2:3. The age of onset varied across childhood, adolescence, and adulthood, with an average age of 29.7 years (ranging from the youngest at 9 years old to the oldest at 61 years old) (Supplementary Materials-Tables S1 and S2). The clinical symptoms were categorized based on the Consensus Mitochondrial Disease Criteria (MDC) scoring system (Table 1), which is an improved consensus system established in 2006 [8]. The highly prevalent clinical manifestations included muscular manifestations (symptoms of proximal muscle weakness, exercise intolerance, ocular muscle paralysis and muscle atrophy in 34.5%, 27.6%, 10.3% and 3.4% of patients, respectively), central nervous system (CNS) manifestations (symptoms of stroke-like episodes, migraine-like headache, cognitive decline and seizure in 31.0%, 24.1%, 20.7% and 20.7% of patients, respectively), and multisystem manifestations (endocrine, visual, digestive and auditory system involvement in 41.4%, 31.0%, 29.0% and 27.6% of patients, respectively). Among the 29 individuals with MELAS syndrome, limb convulsions and proximal muscle weakness were identified as the primary early symptoms (Table 1). Figure 1 and Table 2 provided a concise overview of the clinical features and diagnostic examination results for 29 patients diagnosed with MELAS syndrome. According to the MDC scores, 27.6% of the patients with scores higher than 8 points were classified as having “definite” mitochondrial disorders, and 51.7% of the patients with scores between 5 and 7 points were classified as having “probable” mitochondrial disorders. We followed up 29 individuals diagnosed with MELAS syndrome by telephone, and the findings revealed that 12 patients had expired. Four patients died within five years after being diagnosed with MELAS, whereas eight died within five to ten years. In addition, 17 patients with MELAS syndrome have survived to date (Fig. 2). More detailed demographic, neuroradiological, morphological, and genetic data are provided in the Supplementary Materials-Table S1.

**Table 1:** Classified specific manifestations of 29 patients with MELAS syndrome

Clinical manifestation	n	%
*A. Initial symptoms*		
Limb convulsions	6	20.7
Proximal muscle weakness	5	17.2
Tinnitus or hearing impairment	4	13.8
Fever	3	10.3
Ptosis	2	6.9
Speech disorder	2	6.9
Seizure-like episodes	2	6.9
Decreased consciousness	2	6.9
Migraine-like headache	1	3.4
Blurry vision	1	3.4
Mental abnormalities	1	3.4
*B. Muscular manifestation*		
Proximal muscle weakness	10	34.5
Exercise intolerance	8	27.6
Ocular muscle paralysis	3	10.3
Muscle atrophy	1	3.4
*C. CNS manifestation*		
Stroke-like episodes	9	31.0
Migraine-like headache	7	24.1
Cognitive decline	6	20.7
Seizure	6	20.7
Language impairment	6	20.7
Decreased consciousness	6	20.7
Dizziness	5	17.2
Limbs numbness	4	13.8
Developmental delay	4	13.8
Extrapyramidal signs	1	3.4
*D. Multisystem manifestation*		
Endocrine system	12	41.4
Digestive system	9	31.0
Visual system	8	29.0
Auditory system	8	27.6
Immune system	7	24.1
Mental system	6	20.7
Cardiovascular system	2	6.9
Urinary system	1	3.4
Sensory system	1	3.4
E. MDC score(points) *		
8–12 points	8	27.6
5–7 points	15	51.7
2–4 points	6	20.7
1 point	0	0

^*^MDC score: consensus mitochondrial disease criteria (MDC) score [8], Score 1: mitochondrial disorder unlikely; score 2–4: possible mitochondrial disorder; score 5–7: probable mitochondrial disorder; score 8–12: definite mitochondrial disorder

**Table 2:** Diagnostic examination results in 29 Patients with MELAS Syndrome

Diagnostic examination	Results	n/N*
*Metabolic studies*		
Blood lactate	Elevated lactate concentration	10/12
*Imaging studies*		
^1^H-MRS	Abnormal Lactate Peak(Elevated lactate)	6/9
	Decreased N-Acetylaspartate (NAA)	8/9
	lower NAA with higher lactate	5/9
^31^P-MRS	Elevation of Pi/PCr compared to normal values in rest	1/4
	Significant increase in post-exercise Pi/PCr	4/4
	Moderate decrease in Pi/PCr during the recovery phase	4/4
*Morphology*		
Muscle biopsy	Ragged red fibers (RRFs)	14/16
	Ragged blue fibers (RBFs)	9/16
	COX-positive fibers	10/16
	COX-negative fibers	12/16
	SDH-positive blood vessels	9/16

^*^ “N” represents the total number of cases of a certain diagnostic examination, and “n” represents the number of cases with the results of the certain examination

### Blood lactate concentration

In the present study, lactate level testing was conducted on 12 patients at rest and just after exercise (two time points). The normal range of blood lactate is 0.7–2.1 mmol/L. Ten patients presented elevated lactate values (hyperlactataemia) at rest. In addition, three patients demonstrated a substantial increase in the lactate concentration immediately after exercise, exceeding three times the resting/baseline level. Nine patients exhibited moderately elevated lactate levels (no more than threefold elevation) just following exercise.

### Neuroimaging features in MRI/MRS

**Fig. 3:**
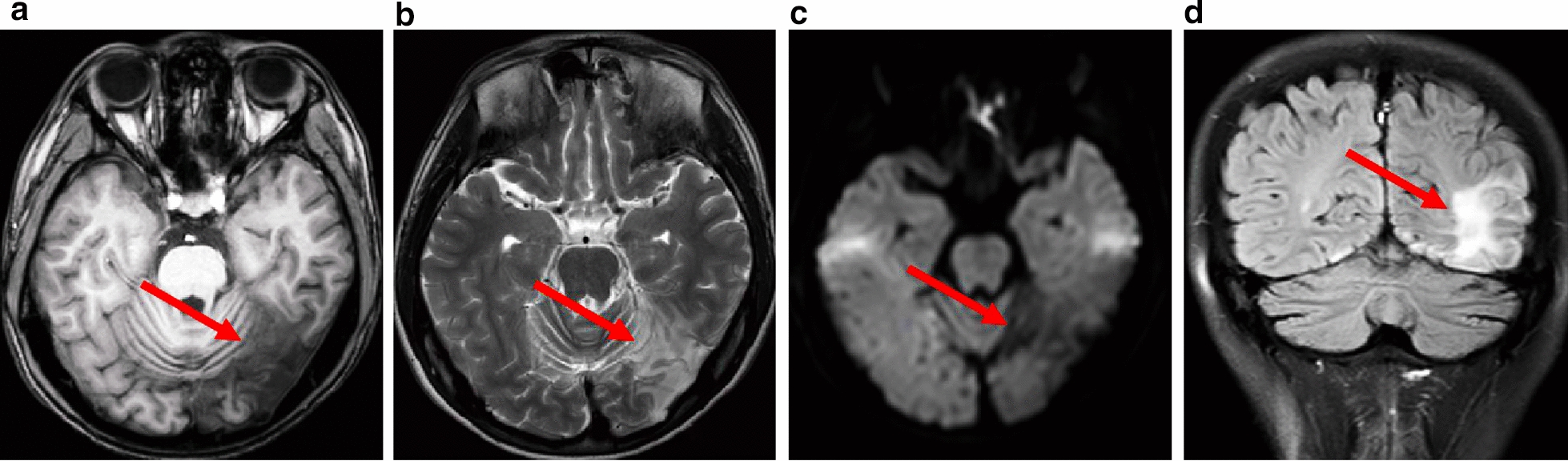
Brain MR image of a patient with MELAS syndrome. Neuroimaging revealed that the left occipital location did not align with the vascular territories affected by ischaemic stroke during stroke-like episodes: patchy T1WI hypointensity (**A**) and T2WI hyperintensity (**B**). The DWI sequence exhibited scattered slight diffusion limitations, with a scattered strip with a high signal in a gyriform pattern (**C**). The FLAIR sequence revealed occipital subcortical hyperintensity (**D**)

**Fig. 4:**
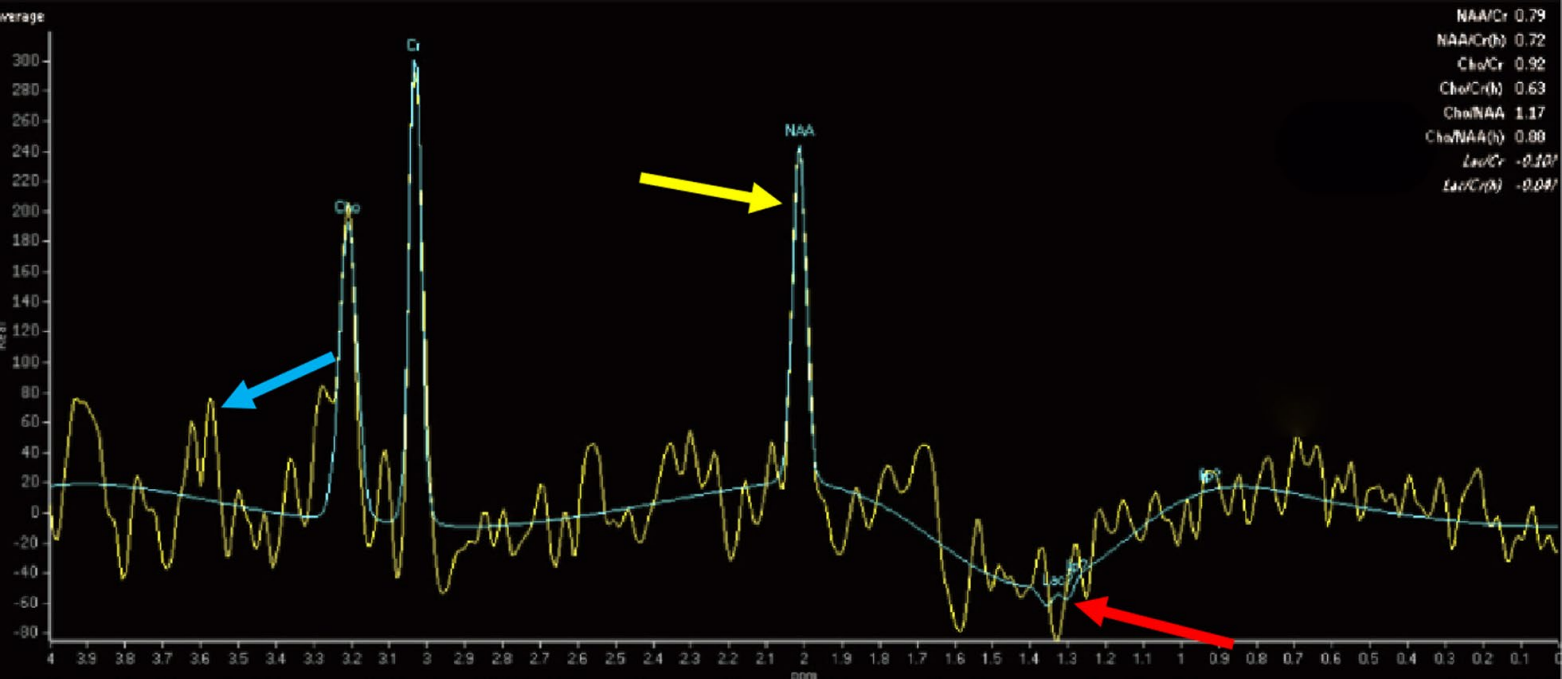
Brain ^1^H-MRS image of a patient with MELAS syndrome. The ^1^H-MRS analysis revealed an inverted lactate double peak at 1.33 ppm, indicating the presence of accumulated lactate within the lesion (red arrow). In addition, the concentration of N-acetylaspartate (NAA) was reduced at 2.02 ppm (yellow arrow), with an NAA/Cr ratio of 0.79 (the normal range for NAA/Cr is 1.8–2.2), which indicates impaired neuronal function. There was a mild increase in the mI/Cr ratio at 3.56 ppm (blue arrow), but this increase was not statistically significant

The MRI scans of 21 MELAS patients showed T2WI and FLAIR hyperintensity, mostly in the parietal (47.6%), temporal (28.6%), occipital (23.8%) and frontal (19.0%) cortices (Fig. 3). These areas did not align with the vascular territories associated with ischaemic stroke. Subcortical deep brain areas, such as the basal ganglia (28.6%), cerebellum (19.0%), brainstem (4.8%), and ventricle (4.8%), may be preferentially involved. ^1^H-MRS is a noninvasive method for assessing metabolite changes in brain tissue in vivo. Two main metabolic indices have been identified: NAA, a biomarker of neuronal integrity, and Lac, a biomarker of mitochondrial failure and anaerobic energy metabolism. In this study, ^1^H-MRS was used to assess the NAA and Lac cerebral metabolic indices in 9 MELAS cases. Approximately 88.9% of patients had a decreased NAA level (normal ratio: 1.8–2.2), and 66.7% of patients had an abnormally elevated lactate peak (normal ratio: 0–0.2). A combination of lower NAA/Cr and higher Lac/Cr was demonstrated in 55.6% of patients (Fig. 4) when creatine was used as an internal control. Our results indicate that mitochondrial dysfunction might lead to impaired neuronal viability (NAA decline) and anaerobic glycolysis (Lac accumulation) [9].

**Fig. 5:**
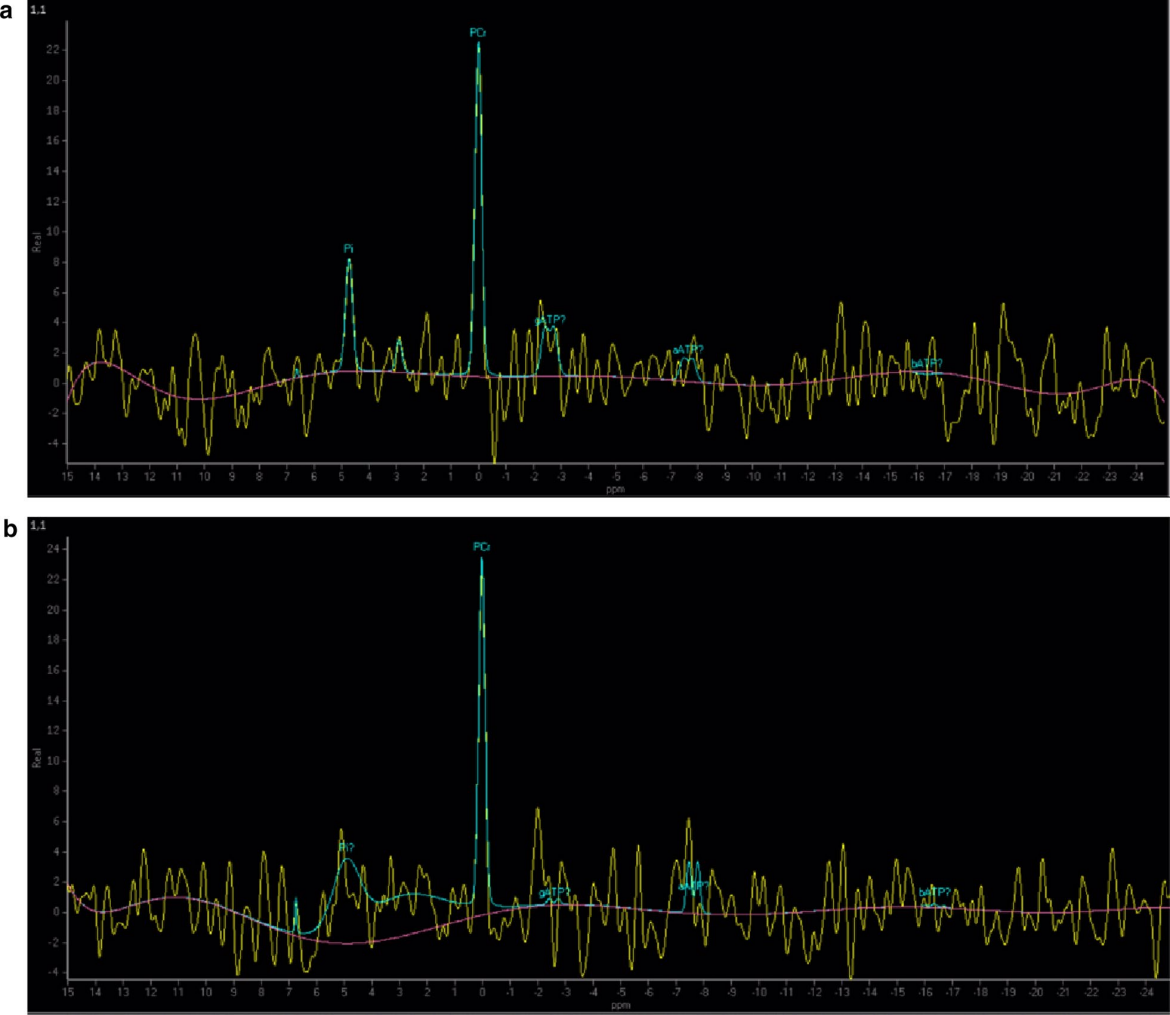
Quadriceps muscle ^31^P-MRS image of a patient with MELAS syndrome. Immediately after exercise, the Pi levels rapidly increased and the PCr levels decreased, leading to a remarkable increase in the Pi/PCr ratio (**A**). During the recovery period after exercise, PCr levels gradually returned to their resting phase level, accompanied by a slow decline in the Pi/PCr ratio (**B**)

^31^P-MRS is considered a highly sensitive and precise technique for evaluating mitochondrial energy and ATP generation in skeletal muscle [6]. The quadriceps muscle was selected because of its prominent high-energy phosphate PCr and ATP peaks. A serial ^31^P-MRS procedure was conducted on 4 MELAS patients to assess their condition during rest, immediately after activity, and during the recovery phase (5 min after exercise). One patient presented an elevated Pi/PCr ratio during the resting state (normal ratio: 0.1–0.2). In addition, all four patients in our study presented a notable increase in the Pi/PCr ratio just after exercise. During the recovery phase, the Pi/PCr ratio slowly decreased and then gradually returned to the resting/baseline value during the remaining period (Fig. 5). These observations suggest impaired high-energy phosphate transfer in proximal muscles and mitochondrial dysfunction in MELAS.

### Genetic testing

All patients underwent genetic testing (Supplementary Materials-Table S1). The m.3243A > G variant was identified in twenty-five patients. Three patients in our cohort had a family history of the m.3243A > G variant, including the daughter, mother and grandmother. Genetic tests were obtained from their urine samples and the variant ratios of m.3243A > G were 89.3% (daughter), 66.7% (mother) and 42.5% (grandmother). The daughter exhibited limb convulsion, her mother exhibited visual and auditory impairment, and her grandmother suffered from a slight headache and tinnitus due to a relatively low proportion of mutants. 

Additionally, several uncommon variants were observed: (1) A 29-year-old male patient presented with proximal muscle weakness and headache. Next-generation sequencing of full-length mtDNA (point variants, deletions and repetitive variants) revealed a likely pathogenic variant, m.5628 T > C, with a frequency of 99.38% (variant frequency = variable base/(variable base + reference base)). No additional mtDNA deletions or repetitive variants were identified. (2) A 13-year-old male patient presented with myopathic facial features, blurred vision, limb weakness, short stature, exercise intolerance, and bilateral ptosis. Next-generation mtDNA sequencing revealed a single large-scale 7601-bp deletion from the 6352–13952 locus (m.6352-13952del). 3) Two of our patients displayed different 9-bp deletion variants combined with m.3243A > G: one 52-year-old female had a 9-bp mtDNA deletion from the 8281–8289 locus (m.8281-8289del) and presented with seizure-like episodes, migraine-like headache, and dizziness. The other 40-year-old female presented a 10-bp mtDNA deletion from the 8281–8290 locus with an additional insertion of base “A” (m.8281-8290del insA), and presented symptoms such as fever, nausea, vomiting, and psychiatric abnormalities.

### Histological examinations

**Fig. 6:**
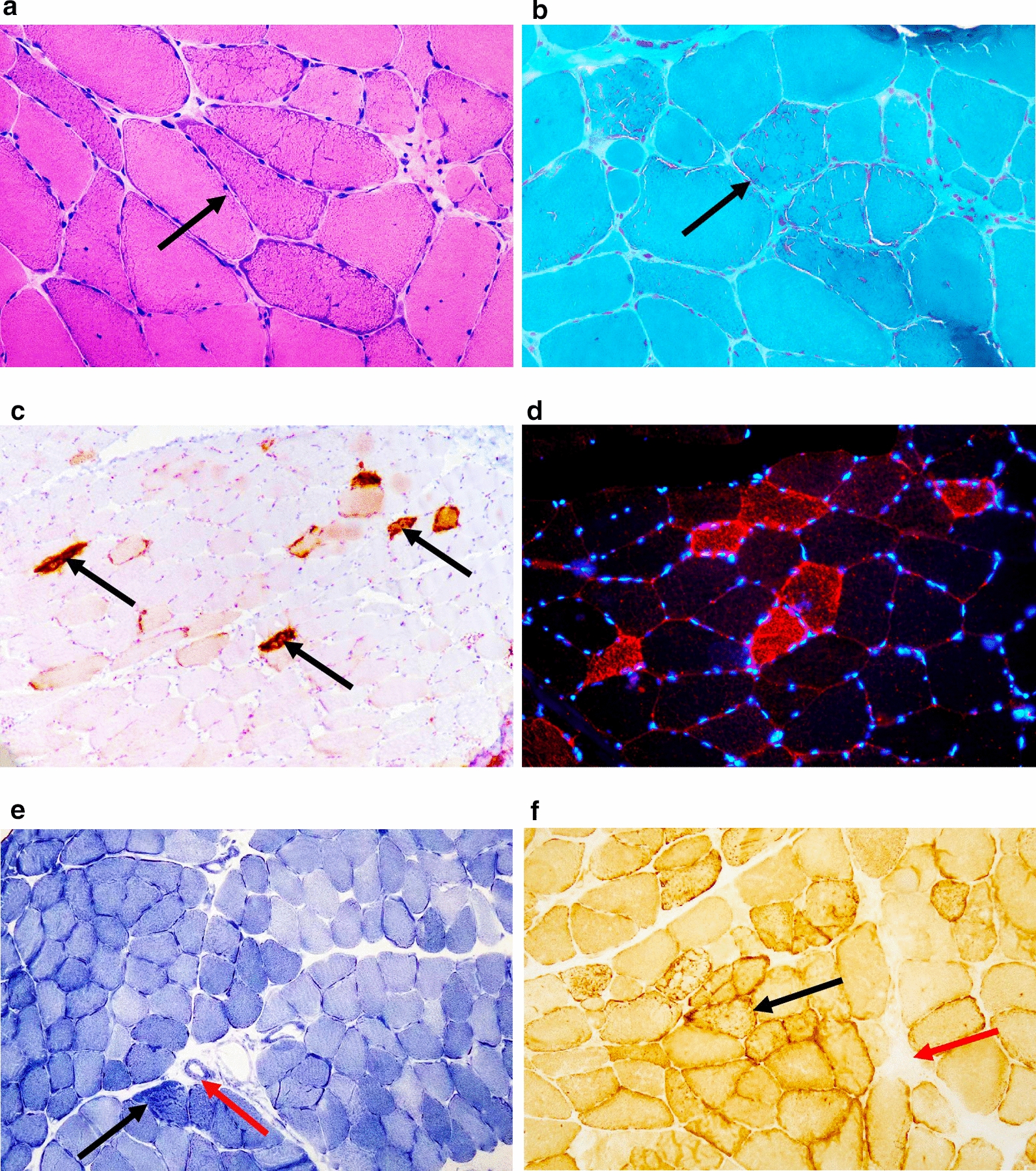
Muscle biopsy from the biceps brachii and immunohistochemical/ immunofluorescence staining of a patient with MELAS syndrome due to m.3243 A > G. H&E staining revealed the presence of several RRFs with basophilic sarcoplasmic masses that were irregular in shape (**A**. 400 × magnification), indicating abnormally proliferated mitochondria. MGT staining revealed that these RRFs were intensely red in colour, especially in the subsarcolemmal zones but also throughout the fibres (**B** 400 × magnification). RRFs showed notable positivity in AMA staining (**C**. 200 × magnification) in the paraffin-embedded section and in the frozen section (**D** 400 × magnification). SDH staining revealed the appearance of RBFs (black arrow) and SSVs (red arrow) (**E** 200 × magnification). COX staining revealed COX-positive (black arrow) and COX-deficient fibres (red arrow) (**F** 200 × magnification)

The investigation of mitochondria in muscle tissue has become crucial for patients who are suspected of having mitochondrial encephalomyopathy. In the present study, muscle biopsies were performed on 16 patients. The biceps brachii muscle (5 patients) and the lateral thigh muscle (11 patients) were selected. The morphological hallmark of mitochondrial diseases is mitochondrial myopathy, which is characterized by abnormal mitochondrial proliferation and/or enzyme histochemical evidence of oxidative phosphorylation (OXPHOS) deficiency. In our 14 patients, abnormal mitochondrial proliferation was observed as RRFs via H&E and MGT staining, mostly in the subsarcolemmal region but also distributed throughout the fibre. Immunohistochemical staining was performed on samples from 14 patients using AMA antibody to assess aberrant mitochondrial proliferation (RRFs). As shown in Fig. 6, AMA staining was positive in RRFs because of the significant increase in the number of proliferated mitochondria. Moreover, immunofluorescence staining was used as a supplementary method to identify AMA antibodies in one patient, whereas DAPI was utilized as a blue-coloured marker for the nucleus. The results further revealed that the AMA antibody effectively increased the number of RRFs in muscle fibres caused by abnormally proliferated mitochondria.

Staining for SDH and COX is crucial because it helps to identify scattered muscle fibres with increased SDH activity (RBFs) and COX deficiency, which are indicative of defective OXPHOS (COX-deficient fibres can be visualized by blue staining). In the present study, nine patients presented RBFs with a distribution similar to that of RRFs, and twelve patients presented COX-negative fibres due to mtDNA defects. Moreover, strongly SDH-reactive blood vessels have been interpreted as a sign of MELAS [10]. Within our cohort, 9 patients presented with small vessels with positive staining for SDH (SDH staining of small vessels, SSV), indicating the presence of mitochondrial vasculopathy. Somewhat specific to MELAS, an increase in the activity of the COX enzyme is common. In our study, more than half of the patients presented with local enzymatic overexpression of COX, which is considered a distinctive pathological feature of MELAS (Fig. 6). Occasionally, glycogen and lipids accumulate in muscle fibres alone with proliferated mitochondria. Among our patients, three had positive PAS staining, indicating the presence of glycogen deposition. Additionally, one patient showed positive Oil Red O staining, suggesting an increase in lipid droplets.

**Fig. 7:**
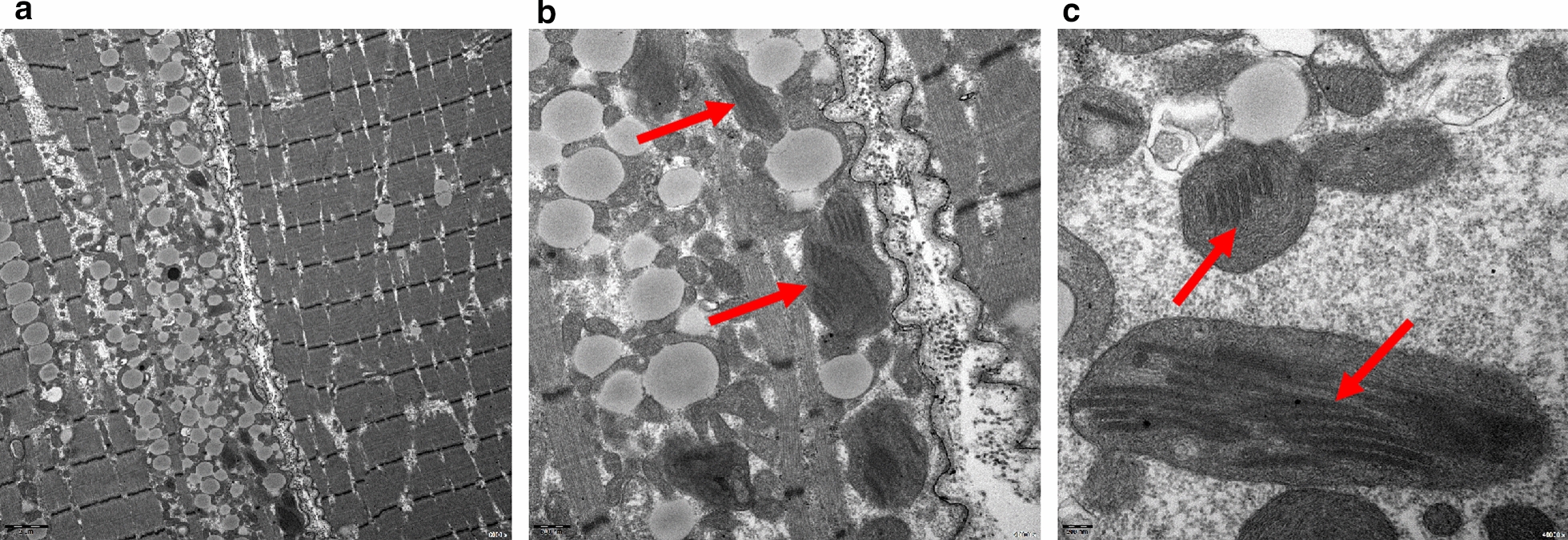
Electron microscopy examination of a patient with MELAS syndrome due to m.3243 A > G. Electron micrographs illustrated the buildup of lipid droplets and the presence of abnormal mitochondria of various sizes between myofibrils in the subsarcolemmal zone (**A** 6000 × magnification). The red arrow highlights different forms of mitochondrial abnormalities, such as giant, structurally abnormal mitochondria and paracrystalline inclusions (**B** 20,000 × magnification, **C** 40,000 × magnification)

To observe the ultrastructures of aberrant mitochondria, electron microscopy was performed in a total of 10 patients. Electron micrographs revealed various structural abnormalities in RRFs, mostly paracrystalline inclusions that manifested with a parking lot-like appearance (Fig. 7). Other changes to the mitochondrion may include enlargement and abnormal arrangements of the cristae.

**Fig. 8:**
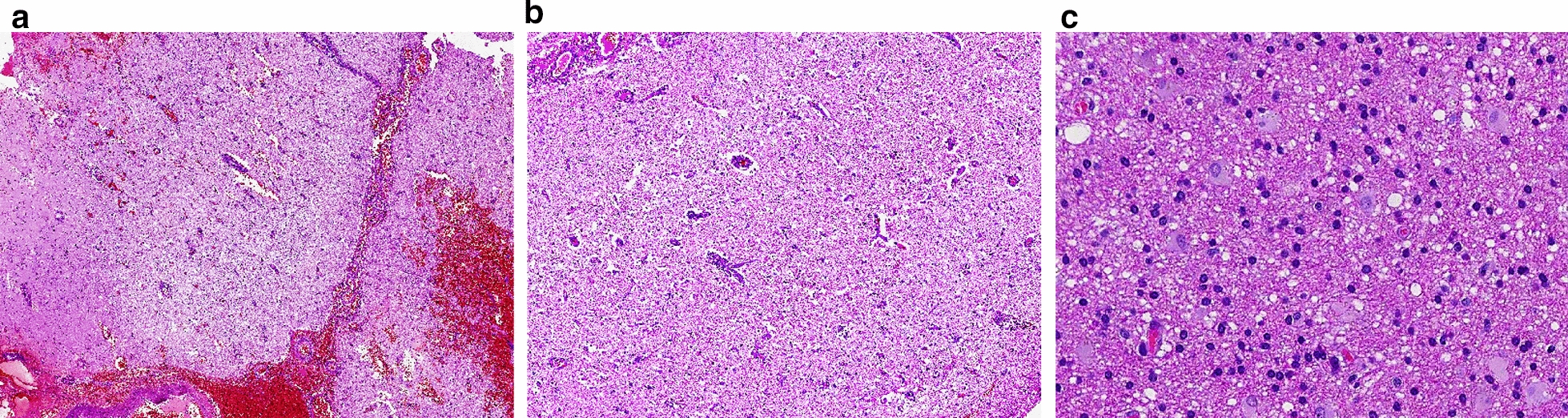
Brain lesion in a patient with MELAS syndrome in the left frontotemporal lobe. The small blood vessels showed dilation and congestion on the surface of the meninges (**A** 40 × magnification). A magnified view of a portion of the infarct reveals the proliferation of glial cells, vacuolization of the adjacent neuropil, and prominent capillaries (**B** 100 × magnification). Note the neuronal swelling of the cerebral parenchyma at higher magnification (**C** 200 × magnification), which represented neuronal degeneration to a certain extent [11]

A suspected patient with a left frontotemporal lobe lesion underwent a surgical procedure to excise a portion of the brain tissue due to an initial misdiagnosis of the neoplasm. The primary clinical symptoms of this patient were speech disorders, memory impairment and headache. This brain tissue was examined via paraffin-embedded sections. The subsequent genetic test revealed a point variant of m.3243A > G that led to a definitive diagnosis. Neuropathological examination revealed histologic abnormalities similar to those of small subacute infarcts in patients with ischaemic and hypoxic encephalopathy. Early activation of microglia and the subsequent formation of small cerebral vessels are responses to tissue damage. This patient’s tissue showed evidence of neovascularization and endothelial proliferation. Neuronal eosinophilia and neuronal loss may persist at this stage but are variable in extent and timing (Fig. 8).

**Fig. 9:**
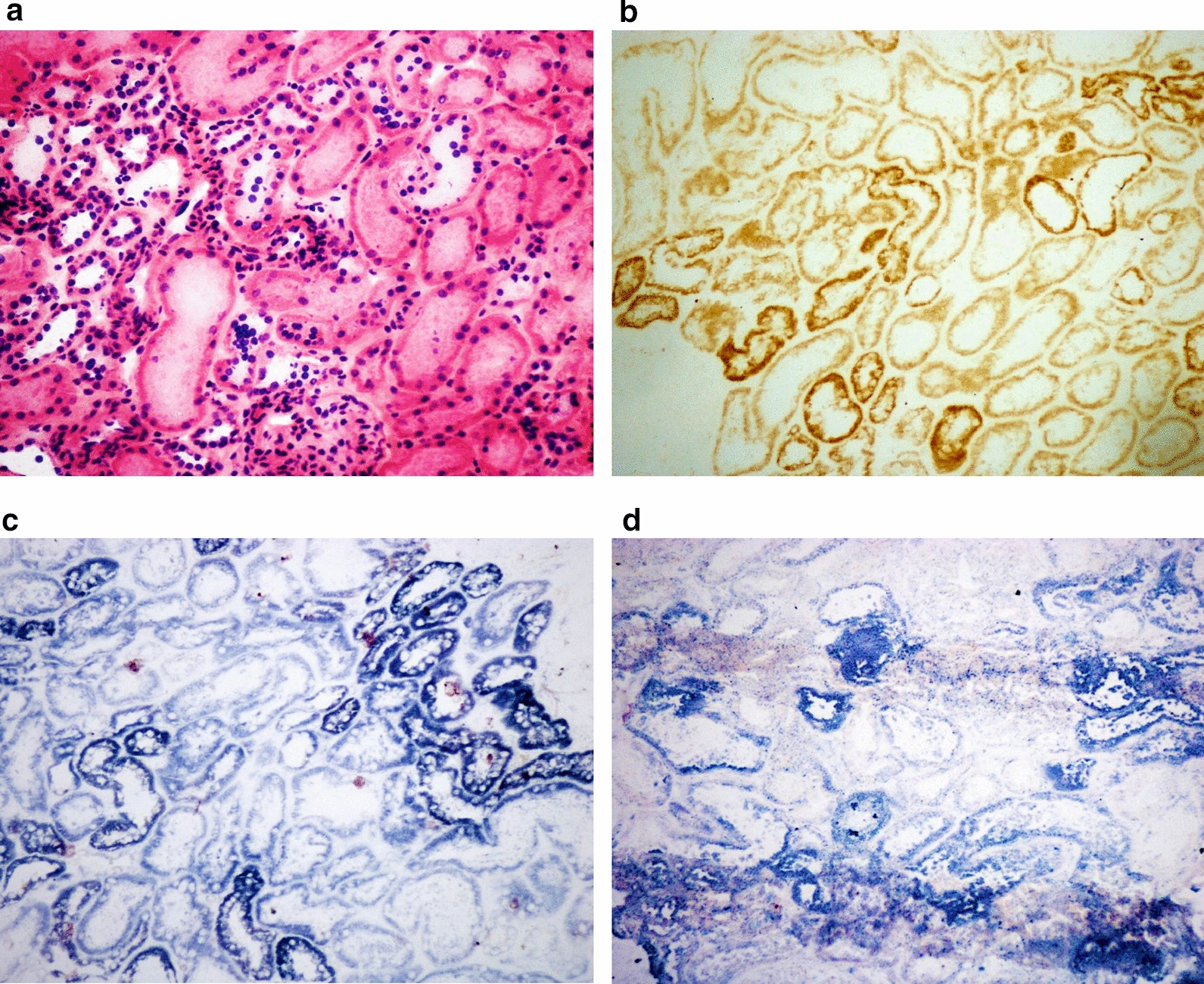
Renal biopsy of a patient with MELAS syndrome. The kidney samples were processed into frozen sections and were examined via COX, SDH and NADH enzyme activity staining. H&E staining (**A** 20 × magnification) revealed the structure of the renal glomerulus and tubules, which appeared basically normal. Enzymatic staining suggested decreased mitochondrial activity due to defective OXPHOS. Serial sections demonstrate decreased activity of COX (**B** 20 × magnification), SDH (**C** 20 × magnification), and NADH (**D** 20 × magnification) in renal tubules

A suspected patient with impaired kidney function characterized by the presence of proteinuria, underwent renal biopsy as a result of an initial incorrect diagnosis of nephropathy. The slight clinical features include muscle weakness, exercise intolerance, and mild headache. The variant was identified as m.3243A > G by further genetic analysis. Although H&E staining showed that the structure of renal glomeruli and tubules appeared normal, with no atrophy, dilatation, or degeneration, enzyme histochemical alterations were observed via COX, SDH and NADH staining which revealed decreased enzyme activity due to defective OXPHOS (Fig. 9).

## Discussion

The present study summarizes the clinical characteristics, MRI/MRS imaging, histopathological features and molecular gene variants of MELAS syndrome. We focused on the central nervous system and skeletal muscle, which are involved mainly because of their high energy requirements and insufficient energy supply [3, 12]. Enzyme histochemistry, electron microscopy, mtDNA analysis, and MRI/MRS techniques are commonly used to analyse proliferated abnormal mitochondria in muscle or brain tissue to accurately diagnose MELAS. Muscle biopsy is typically performed on patients suspected of having mitochondrial myopathy, particularly those with chronic muscle weakness and exercise intolerance.

The pathological processes of MELAS involve abnormalities in mitochondrial metabolic function and deficiencies in enzyme activities. Stroke-like episodes are a prominent clinical feature of MELAS syndrome, manifesting as reversible aphasia, cortical blindness, altered mental status, and migraine [3, 13], which ultimately lead to progressive neurological impairment [3, 7, 14]. Some MELAS patients may experience cognitive decline due to alterations in cerebral blood flow, even if they do not have obvious stroke-like episodes [3, 14]. Because stroke-like episodes can mimic acute neurological deficits caused by subacute cerebral infarction, MRI is important for differential diagnosis, particularly when the location does not correspond to typical ischaemic stroke regions in children or adolescents [15]. The cerebral pathological alterations observed in our MELAS patients were similar to those observed in subacute ischaemic stroke patients and were characterized by the presence of activated glial cells, proliferated capillaries and hypoxic neurons. The remaining white matter showed a mild spongiform appearance, with no apparent cerebral oedema. Although these cerebral abnormalities lack specificity, the presence of infarct-like lesions, neuronal eosinophilia, and/or spongiform changes could suggest a diagnosis of MELAS [16–18], in an appropriate clinical setting.

Abnormal cerebral mitochondrial metabolism may lead to migraine and stroke-like episodes under conditions of increased metabolic demand. On the one hand, mitochondrial vasculopathy may partially explain the specific aetiology of stroke-like episodes. Compared with those of larger blood arteries, the arterioles of the pia mater are more significantly affected by aberrant mitochondrial proliferation [19]. The fundamental cause of vascular alterations and the underlying basis of brain lesions in MELAS may be attributed to the primary dysfunction of mitochondria in cerebral vascular smooth muscle and endothelial cells [19, 20]. This dysfunction, in turn, could lead to the disruption of microcirculation and subsequent damage to brain tissue [21]. In addition, our MELAS patients presented both COX-positive and COX-negative muscle cells, indicating elevated respiratory enzyme activity at the local level [3], but a general decrease in enzyme activity overall. The overall COX expression within small cerebral arteries is postulated to be reduced, although localized COX activity may increase, a phenomenon that is similar to that observed in muscle biopsies. The mechanisms underlying the inherent contradiction between residual enzyme activity and stroke-like episodes, however, remain unclear. A possible explanation for this finding might be that excessive COX can bind to nitrogen monoxide (NO) in cerebral small vessels. NO plays an important role in mediating smooth muscle vasodilation. Therefore, excessive elevation of COX levels could lead to a relative shortage of circulating NO that causes vasodilation retardation and local hyperperfusion [6]. Moreover, a marked increase in the local lactate concentration could impede endothelial cell function and increase vascular permeability, leading to vasogenic oedema and local hyperperfusion [22, 23]. Hence, the increased residual COX activity and local lactate within the mitochondrial vasculopathy may be associated with the occurrence of migraine and stroke-like episodes in MELAS.

The infarct-like lesions in the occipital, parietal, and temporal cortical regions (may be multifocal) or subcortical deep brain structures, including the basal ganglia and brainstem, are not confined to traditional vascular distributions. This phenomenon suggests that stroke-like episodes may be caused by metabolic abnormalities rather than vascular abnormalities [24]. On the other hand, the specific stroke-like episodes may be partially attributed to mitochondrial neuropathy. Mitochondrial neuropathy has the potential to induce neuronal vulnerability triggered by prolonged hyperexcitability of neurons [25]. NAA is considered a marker of neuronal viability. NAA is localized exclusively in neurons and synthesized in neuronal mitochondria. The regulation of NAA content is influenced primarily by the rate of mitochondrial synthesis, and the NAA concentration correlates with neuronal mitochondrial function [26]. Compared with glial cells, neurons are more susceptible to energy deficiencies [27]. The present study revealed that a decreased NAA level was predictive of impaired neuronal viability and malfunction in neuronal mitochondria. Although a tendency towards mild proliferation of glial cells was observed, as indicated by the mI level in the MRS, this difference was not significant. In addition, our MELAS patients demonstrated a decrease in NAA/Cr, which was associated with an increase in the Lac peak. Mitochondrial dysfunction might be a potential cause of elevated lactate levels [28]. The extracellular accumulation of lactate is a consequence of increased anaerobic glycolysis caused by an insufficient oxygen supply to neuronal tissues [29]. Thus, ^1^H-MRS may be used for the early monitoring of brain metabolic changes related to mitochondrial neuropathy, and it may serve an indicator for evaluating the severity of stroke-like episodes in MELAS.

In accordance with previous studies, the most common variant in MELAS is m.3243A > G tRNA^Leu^-(UUR), accounting for approximately 87% of mutations in our cohort. Because the variant rate is higher in urinary cells than in peripheral blood [30], urine samples could be a preferred choice for mtDNA detection. MELAS is widely recognized as a heteroplasmic and complex subtype of mitochondrial encephalomyopathy. The same gene variant may produce diverse clinical symptoms, whereas different genetic variants may induce similar clinical manifestations [31]. In our study, one family carrying different proportions of m.3243A > G variants presented various clinical manifestations (clinical heterogeneity). In general, the variant ratio (also known as the variant load) may be somewhat correlated with the severity of the clinical neurological symptoms. Compared with the daughter, the mother and grandmother in our cohort presented modest symptoms despite carrying mutant mtDNA that was maternally inherited. This differential presentation is due to the heteroplasmy state of mtDNA, in which mutant and wild types coexist, and the proportion of mutant types is relatively low in mother and grandmother [12].

In addition, the presence of different variant sites in MELAS could indicate similar clinical manifestations (genetic heteroplasmy). Our study identified several infrequent variants that serve as complements to m.3243A > G (MELAS-non m.3243A > G). (1) The m.5628 T > C variant was reported as a new variant in the mitochondrial tRNA^Ala^ that satisfied the criteria for pathogenicity [32]. Nucleotide position 5628 is a highly conserved nucleotide that was not found in the control population, and its substitution could impact the function of tRNA^Ala^. The T–C transition disrupts a conserved A-U base pair within the anticodon stem of the tRNA^Ala^ two-dimensional structure. This substitution results in the loss of a hybrid pair, leading to the generation of nine unmatched nucleotides [32]. As a consequence of this altered tertiary structure, the formation of the stem and loop is unstable, which may impair the function of tRNA^Ala^ and affect intramitochondria protein synthesis. However, the clinical characteristics described in the literature differed from those reported in our patient, who presented with notable proximal muscular weakness and migraine. In contrast to the previously reported 62-year-old elderly female, who presented with late-onset chronic progressive external ophthalmoplegia, our young male patients with the same m.5628 T > C variant did not present symptoms of ophthalmoplegia during the course of the disease or during the follow-up. These differences further suggested that the same variant site could give rise to diverse clinical phenotypes. (2) Our study revealed a single large-scale 7601-bp deletion of m.6352-13952del (heteroplasmic state). The clinical manifestations and pathological RRFs provide evidence for the diagnosis of MELAS syndrome, at least in part. In addition, the large-scale deletion from m.6352 to 13,952 may impair the function of several mtDNA genes and tRNA, similar to the m.3243A > G variant, suggesting the pathogenicity of the m.6352-13952del variant. Mitochondrially encoded NADH dehydrogenase (*MT-ND*) is the second most common pathogenic mitochondrial gene in MELAS, following *MT-TL1* which is caused by m.3243A > G [33]. The affected mtDNA genes and tRNAs of m.6352-13952del include the following (see Mitomap): *MT-ND*, mitochondrially encoded tRNA leucine (tRNA-Leu), and mitochondrially encoded cytochrome c oxidase (*MT-COX)* [33, 34]. Thus, based on these pathogenic MT genes and MT-tRNAs, the variant of m.6352-13952del could be classified into MELAS-non m.3243A > G. (3) Two of our patients had different types of 9 bp deletions that combined with pathogenic variants of m.3243A > G. Although m.8281_8289del or mt.8281-8290del insA may be a genetic polymorphism that occurs in a noncoding region of mtDNA, it is likely associated with the development of mitochondrial encephalomyopathies, such as the MELAS or MERRF subtypes [35], as well as other disorders such as gestational diabetes mellitus (GDM) [36]. Further investigation is needed to determine its possible modulatory role.

This study may have certain limitations. First, our study revealed a brain atrophy rate of approximately 50%, which was lower than the reported proportion of up to 90% reported in previous studies. This discrepancy might be attributed to the relatively small sample size of patients with different age distributions and disease severities. Second, we were unable to provide data on cerebrospinal fluid lactate levels because the majority of patients were unwilling or unable to undergo lumbar puncture. Third, we could not detect brain COX enzyme activity because of the regular fixation of the surgical specimen in 10% formaldehyde, which prevented us from obtaining frozen sections and performing enzymatic staining procedures. Fourth, we were unable to perform further enzymatic and immunostaining because of a shortage of tissue for kidney biopsy.

## Conclusions

In summary, this work provides a comprehensive overview of the clinical and multisystemic pathological features, imaging examinations, and genetic analysis of MELAS syndrome. It has demonstrated clinical diversity and mtDNA heteroplasmy as a rare and complex mitochondrial encephalopathy subtype. Additionally, it identified several infrequent variants that serve as complements to m.3243A > G. The pathological processes of MELAS involve abnormalities in mitochondria (RRFs) and defects in COX enzyme activity. Mitochondrial vasculopathy and mitochondrial neuropathy may be the underlying causes of stroke-like episodes, at least to some extent. MELAS can be diagnosed based on postexercise hyperlactatemia, brain lesions that deviate from the typical vascular distribution of cerebral infarction on MRI, elevated lactate peaks with reduced NAA on spectroscopy, and the presence of pathological RRFs on muscle biopsy.

## Supplementary Information

Additional file 1.

Additional file 2.

## Data Availability

The authors confirm that the demographic and clinical data supporting the findings of this study are available within the article and its supplementary materials. Due to privacy concerns and to protect patient confidentiality, the pathological and genetic data that support the findings of this study are not publicly available. Access to the data may be granted under strict confidentiality agreements upon request. For inquiries related to data access, please contact the corresponding author.

## Abbreviations

^1^H-MRS: Proton magnetic resonance spectroscopy
^31^P-MRS: Phosphorus magnetic resonance spectroscopy
Ala: Alanine
ATP: Adenosine triphosphate
AMA: Anti mitochondrion antibody
bp: Base pair
CNS: Central nervous system
COX: Cytochrome c oxidase
Cr: Creatine
DAPI: 4',6-Diamidino-2-phenylindole
DWI: Diffusion-weighted imaging
EWS: Extended workspace method
FLAIR: Fluid-attenuated inversion recovery
FOV: Field of view
GDM: Gestational diabetes mellitus
H&E: Hematoxylin and eosin
Lac: Lactate
Leu: Leucine
MDC: Mitochondrial disease criteria
MELAS: Mitochondrial encephalomyopathy, lactic acidosis, and stroke-like episodes
MERRF: Myoclonus epilepsy with ragged-red fibers
MGT: Modified Gomori trichrome
mI: Myo-Inositol
MRI: Magnetic resonance imaging
MRS: Magnetic resonance spectroscopy
*MT-COX*: Mitochondrially encoded cytochrome c oxidase
mtDNA: Mitochondrial DNA
*MT-ND*: Mitochondrially encoded NADH dehydrogenase
*MT-TL1*: Mitochondrial Leucine tRNA-1
NAA: N-acetylaspartate
NADH: Nicotinamide adenine dinucleotide hydrogen
nDNA: Nucleic DNA
NO: Nitrogen monoxide
OXPHOS: Oxidative phosphorylation
PAS: Periodic acid-Schiff
PCr: Phosphocreatine
PCR: Polymerase chain reaction
Pi: Inorganic phosphate
PRESS: Point source deconvolution spectrum
RBFs: Ragged blue fibers
RRFs: Ragged red fibers
SDH: Succinate dehydrogenase
SSV: SDH staining of small vessels
T1WI: T1-weighted imaging
T2WI: T2-weighted imaging
tCho: Total choline

## References

[1] Ikeda T, Osaka H, Shimbo H, Tajika M, Yamazaki M, Ueda A, et al. Mitochondrial DNA 3243A>T mutation in a patient with MELAS syndrome. Hum Genome Var. 2018;5:25. 10.1038/s41439-018-0026-6. 30210801 10.1038/s41439-018-0026-6 PMC6123423

[2] Chae HW, Na JH, Kim HS, Lee YM. Mitochondrial diabetes and mitochondrial DNA mutation load in MELAS syndrome. Eur J Endocrinol. 2020;183:505–12. 10.1530/eje-20-0189. 33107434 10.1530/EJE-20-0189

[3] El-Hattab AW, Adesina AM, Jones J, Scaglia F. MELAS syndrome: clinical manifestations, pathogenesis, and treatment options. Mol Genet Metab. 2015;116:4–12. 10.1016/j.ymgme.2015.06.004. 26095523 10.1016/j.ymgme.2015.06.004

[4] Seidowsky A, Hoffmann M, Glowacki F, Dhaenens CM, Devaux JP, de Sainte Foy CL, et al. Renal involvement in MELAS syndrome - a series of 5 cases and review of the literature. Clin Nephrol. 2013;80:456–63. 10.5414/cn107063. 22909780 10.5414/CN107063

[5] Saneto RP, Friedman SD, Shaw DW. Neuroimaging of mitochondrial disease. Mitochondrion. 2008;8:396–413. 10.1016/j.mito.2008.05.003. 18590986 10.1016/j.mito.2008.05.003 PMC2600593

[6] Liu AH, Niu FN, Chang LL, Zhang B, Liu Z, Chen JY, et al. High cytochrome c oxidase expression links to severe skeletal energy failure by (31)P-MRS spectroscopy in mitochondrial encephalomyopathy, lactic acidosis, and stroke-like episodes. CNS Neurosci Ther. 2014;20:509–14. 10.1111/cns.12257. 24674659 10.1111/cns.12257 PMC6493073

[7] Yatsuga S, Povalko N, Nishioka J, Katayama K, Kakimoto N, Matsuishi T, et al. MELAS: a nationwide prospective cohort study of 96 patients in Japan. Biochim Biophys Acta. 2012;1820:619–24. 10.1016/j.bbagen.2011.03.015. 21443929 10.1016/j.bbagen.2011.03.015

[8] Morava E, van den Heuvel L, Hol F, de Vries MC, Hogeveen M, Rodenburg RJ, et al. Mitochondrial disease criteria: diagnostic applications in children. Neurology. 2006;67:1823–6. 10.1212/01.wnl.0000244435.27645.54. 17130416 10.1212/01.wnl.0000244435.27645.54

[9] Bianchi MC, Sgandurra G, Tosetti M, Battini R, Cioni G. Brain magnetic resonance in the diagnostic evaluation of mitochondrial encephalopathies. Biosci Rep. 2007;27:69–85. 10.1007/s10540-007-9046-z. 17510789 10.1007/s10540-007-9046-z

[10] Lu Y, Deng J, Zhao Y, Zhang Z, Hong D, Yao S, et al. Patients with MELAS with negative myopathology for characteristic ragged-red fibers. J Neurol Sci. 2020;408:116499. 10.1016/j.jns.2019.116499. 31726383 10.1016/j.jns.2019.116499

[11] Oliveira A, Hodges H, Rezaie P. Excitotoxic lesioning of the rat basal forebrain with S-AMPA: consequent mineralization and associated glial response. Exp Neurol. 2003;179:127–38. 10.1016/s0014-4886(02)00012-2. 12618119 10.1016/s0014-4886(02)00012-2

[12] Tetsuka S, Ogawa T, Hashimoto R, Kato H. Clinical features, pathogenesis, and management of stroke-like episodes due to MELAS. Metab Brain Dis. 2021;36:2181–93. 10.1007/s11011-021-00772-x. 34118021 10.1007/s11011-021-00772-x

[13] Sproule DM, Kaufmann P. Mitochondrial encephalopathy, lactic acidosis, and strokelike episodes: basic concepts, clinical phenotype, and therapeutic management of MELAS syndrome. Ann NY Acad Sci. 2008;1142:133–58. 10.1196/annals.1444.011. 18990125 10.1196/annals.1444.011

[14] Hirano M, Pavlakis SG. Mitochondrial myopathy, encephalopathy, lactic acidosis, and strokelike episodes (MELAS): current concepts. J Child Neurol. 1994;9:4–13. 10.1177/088307389400900102. 8151079 10.1177/088307389400900102

[15] Jameel I, Sreh A, Das P. Recurrent stroke events secondary to a late presentation of mitochondrial encephalomyopathy with lactic acidosis and stroke-like symptoms (MELAS) syndrome. Cureus. 2020;12:e11839. 10.7759/cureus.11839. 33282603 10.7759/cureus.11839 PMC7714735

[16] Mikol J, Polivka M. Mitochondrial encephalomyopathies. Ann Pathol. 2005;25:282–91. 10.1016/s0242-6498(05)80132-4. 16327654 10.1016/s0242-6498(05)80132-4

[17] Melberg A, Akerlund P, Raininko R, Silander HC, Wibom R, Khaled A, et al. Monozygotic twins with MELAS-like syndrome lacking ragged red fibers and lactacidaemia. Acta Neurol Scand. 1996;94:233–41. 10.1111/j.1600-0404.1996.tb07058.x. 8937533 10.1111/j.1600-0404.1996.tb07058.x

[18] Filosto M, Tomelleri G, Tonin P, Scarpelli M, Vattemi G, Rizzuto N, et al. Neuropathology of mitochondrial diseases. Biosci Rep. 2007;27:23–30. 10.1007/s10540-007-9034-3. 17541738 10.1007/s10540-007-9034-3

[19] Lach B, Preston D, Embree G. Mitochondrial angiopathy in cerebral blood vessels of mitochondrial encephalomyopathy. Acta Neuropathol. 1988;76:216. 10.1007/bf00688107. 3407400 10.1007/BF00688107

[20] Kishi M, Yamamura Y, Kurihara T, Fukuhara N, Tsuruta K, Matsukura S, et al. An autopsy case of mitochondrial encephalomyopathy: biochemical and electron microscopic studies of the brain. J Neurol Sci. 1988;86:31–40. 10.1016/0022-510x(88)90005-6. 2844999 10.1016/0022-510x(88)90005-6

[21] Gropen TI, Prohovnik I, Tatemichi TK, Hirano M. Cerebral hyperemia in MELAS. Stroke. 1994;25:1873–6. 10.1161/01.str.25.9.1873. 8073472 10.1161/01.str.25.9.1873

[22] Walcott BP, Edlow BL, Xia Z, Kahle KT, Nahed BV, Schmahmann JD. Steroid responsive A3243G mutation MELAS: clinical and radiographic evidence for regional hyperperfusion leading to neuronal loss. Neurologist. 2012;18:159–70. 10.1097/NRL.0b013e318247bcd8. 22549360 10.1097/NRL.0b013e318247bcd8

[23] Li X, Wang Y, Wang Z, Lu J, Xu Y, Ye J, et al. Comparison of magnetic resonance spectroscopy (MRS) with arterial spin labeling (ASL) in the differentiation between mitochondrial encephalomyopathy, lactic Acidosis, plus stroke-like episodes (MELAS) and acute ischemic stroke (AIS). J Clin Neurosci. 2018;55:65–70. 10.1016/j.jocn.2018.06.015. 29921486 10.1016/j.jocn.2018.06.015

[24] Leonard JV, Schapira AH. Mitochondrial respiratory chain disorders II: neurodegenerative disorders and nuclear gene defects. Lancet. 2000;355:389–94. 10.1016/s0140-6736(99)05226-5. 10665569 10.1016/s0140-6736(99)05226-5

[25] Niu FN, Meng HL, Chang LL, Wu HY, Li WP, Liu RY, et al. Mitochondrial dysfunction and cerebral metabolic abnormalities in patients with mitochondrial encephalomyopathy subtypes: Evidence from proton MR spectroscopy and muscle biopsy. CNS Neurosci Ther. 2017;23:686–97. 10.1111/cns.12714. 28695670 10.1111/cns.12714 PMC6492760

[26] Paslakis G, Träber F, Roberz J, Block W, Jessen F. N-acetyl-aspartate (NAA) as a correlate of pharmacological treatment in psychiatric disorders: a systematic review. Eur Neuropsychopharmacol. 2014;24:1659–75. 10.1016/j.euroneuro.2014.06.004. 25130303 10.1016/j.euroneuro.2014.06.004

[27] Valanne L, Paetau A, Suomalainen A, Ketonen L, Pihko H. Laminar cortical necrosis in MELAS syndrome: MR and neuropathological observations. Neuropediatrics. 1996;27:154–60. 10.1055/s-2007-973767. 8837076 10.1055/s-2007-973767

[28] Shayota BJ. Biomarkers of mitochondrial disorders. Neurotherapeutics. 2024;21:e00325. 10.1016/j.neurot.2024.e00325. 38295557 10.1016/j.neurot.2024.e00325 PMC10903091

[29] Adam C, Paolini L, Gueguen N, Mabilleau G, Preisser L, Blanchard S, et al. Acetoacetate protects macrophages from lactic acidosis-induced mitochondrial dysfunction by metabolic reprograming. Nat Commun. 2021;12:7115. 10.1038/s41467-021-27426-x. 34880237 10.1038/s41467-021-27426-x PMC8655019

[30] Geng X, Zhang Y, Yan J, Chu C, Gao F, Jiang Z, et al. Mitochondrial DNA mutation m.3243A>G is associated with altered mitochondrial function in peripheral blood mononuclear cells, with heteroplasmy levels and with clinical phenotypes. Diabet Med. 2019;36:776–83. 10.1111/dme.13874. 30536471 10.1111/dme.13874

[31] Wang YX, Le WD. Progress in diagnosing mitochondrial myopathy, encephalopathy, lactic acidosis, and stroke-like episodes. Chin Med J (Engl). 2015;128:1820–5. 10.4103/0366-6999.159360. 26112726 10.4103/0366-6999.159360 PMC4733719

[32] Spagnolo M, Tomelleri G, Vattemi G, Filosto M, Rizzuto N, Tonin P. A new mutation in the mitochondrial tRNA(Ala) gene in a patient with ophthalmoplegia and dysphagia. Neuromuscul Disord. 2001;11:481–4. 10.1016/s0960-8966(01)00195-x. 11404121 10.1016/s0960-8966(01)00195-x

[33] Alston CL, Morak M, Reid C, Hargreaves IP, Pope SA, Land JM, et al. A novel mitochondrial MTND5 frameshift mutation causing isolated complex I deficiency, renal failure and myopathy. Neuromuscul Disord. 2010;20:131–5. 10.1016/j.nmd.2009.10.010. 20018511 10.1016/j.nmd.2009.10.010

[34] Wang W, Zhao Y, Xu X, Ma X, Sun Y, Lin Y, et al. A different pattern of clinical, muscle pathology and brain MRI findings in MELAS with mt-ND variants. Ann Clin Transl Neurol. 2023;10:1035–45. 10.1002/acn3.51787. 37221696 10.1002/acn3.51787 PMC10270267

[35] Liu CS, Cheng WL, Chen YY, Ma YS, Pang CY, Wei YH. High prevalence of the COII/tRNA(Lys) intergenic 9-bp deletion in mitochondrial DNA of Taiwanese patients with MELAS or MERRF syndrome. Ann NY Acad Sci. 2005;1042:82–7. 10.1196/annals.1338.058. 15965049 10.1196/annals.1338.058

[36] Khan IA, Shaik NA, Pasupuleti N, Chava S, Jahan P, Hasan Q, et al. Screening of mitochondrial mutations and insertion-deletion polymorphism in gestational diabetes mellitus in the Asian Indian population. Saudi J Biol Sci. 2015;22:243–8. 10.1016/j.sjbs.2014.11.001. 25972744 10.1016/j.sjbs.2014.11.001 PMC4423658

