# Supplementary material for: Multisystem clinicopathologic and genetic analysis of MELAS
Source: Orphanet J Rare Dis. 2024 Dec 24;19:487. doi: 10.1186/s13023-024-03511-4 (PMC11668008; doi:10.1186/s13023-024-03511-4)
Supplement: Supplementary file 2 — Additional file 2. [file 13023_2024_3511_MOESM2_ESM.docx]

**Table S2 Demographic description of 29 Patients with MELAS Syndrome**

| **Number of cases** | **Male** | | **Female** | | **Age of onset** | | **MDC score** | |
| --- | --- | --- | --- | --- | --- | --- | --- | --- |
| N | n | % | n | % | mean | SD | mean | SD |
| 29 | 12 | 41.4 | 17 | 58.6 | 29.7 | 2.5 | 6.6 | 0.3 |

**Note:** Abbreviation: MDC score: consensus mitochondrial disease criteria (MDC) score.
